# Analysis of the Structural, Chemical, and Mechanical Characteristics of Polyurethane Foam Infused with Waste from Thermal Processing

**DOI:** 10.3390/ma18061327

**Published:** 2025-03-17

**Authors:** Anna Magiera, Monika Kuźnia, Wojciech Jerzak

**Affiliations:** Department of Heat Engineering and Environment Protection, Faculty of Metals Engineering and Industrial Computer Science, AGH University of Krakow, al. A. Mickiewicza 30, 30-059 Krakow, Poland; kuznia@agh.edu.pl (M.K.); wjerzak@agh.edu.pl (W.J.)

**Keywords:** rigid foam, polyurethane, fly ash, fluidized-bed combustion, microspheres, composite material, waste

## Abstract

The continuous generation of agricultural, industrial, and urban waste necessitates effective waste management strategies. One promising approach is incorporating these residues as fillers in polymer composites. This study investigated the influence of coal processing-derived fillers, specifically microspheres and fluidized-bed combustion fly ash, on the structure and properties of composite rigid polyurethane foam. Polyurethane foams were produced through manual mixing and casting, with composite foams containing a combination of 5% microspheres and 5–15% fly ash by weight. The analysis of the samples investigated their structural, thermal, and mechanical properties. The samples consistently displayed predominantly pentagonal, regularly shaped cells. Infrared spectroscopy revealed no observable chemical bonding between the matrix and filler materials. Mechanical analysis was performed to evaluate the materials’ characteristics, revealing significant variations in compressive strength and Young’s modulus values. The results indicate that the addition of fillers did not impact the cellular and chemical composition of the polyurethane matrix. Furthermore, the composite material specimens were subjected to accelerated aging in a laboratory dryer and outdoor exposure in order to assess their thermal stability. This analysis revealed notable alterations in both the cellular composition and mechanical properties of the composite foam materials.

## 1. Introduction

Rigid polyurethane foam (RPUF) has emerged as a highly desirable material for thermal insulation applications due to its favorable characteristics, such as low thermal conductivity, high dimensional stability, low density, and resistance to chemicals and biological factors [1,2]. The increasing demand for polyurethane in diverse industries, including building insulation, food packaging, automotive, medical applications, aviation, and furniture [3,4], can be attributed to the rising energy costs and growing awareness about energy conservation [5]. Furthermore, the properties of the foam can be tailored by incorporating various fillers to develop novel composite materials, allowing for the creation of unique and customized products to meet specific industry needs. The ability to modify and enhance the properties of polyurethane foam through the addition of fillers has led to its widespread adoption across a variety of industries. This flexibility allows manufacturers to create specialized products that cater to the unique requirements of each sector, from insulation for buildings to packaging for delicate goods. The versatility of polyurethane foam, combined with the potential for customization through filler incorporation, has made it an increasingly attractive material choice for a wide range of applications, and it can be adjusted by incorporating fillers to create new composite materials [6,7,8].

Coal combustion for power and heat generation in many countries worldwide produces various combustion by-products, with fly ash being a prominent example [9]. The coal combustion process yields two distinct types of fly ash: one from pulverized coal combustion at high temperatures ranging from 1200 to 1500 °C [10] and another from fluidized bed combustion at temperatures around 800–900 °C [11]. Despite both being classified as waste, these two fly ash types exhibit considerable differences due to their distinct origins. Fly ash composition varies significantly based on raw material and combustion process parameters, with pulverized coal ash (PCC) generally containing fine, glassy spheres and fluidized bed ash (FBC) having more unburned carbon, calcium, and sulfur [12].

The increasing prevalence of fluidized bed combustion fly ash as a byproduct is linked to the shift from traditional pulverized coal boilers towards cleaner coal combustion technologies, such as fluidized bed boilers [13]. This shift is aimed at reducing emissions of harmful pollutants, including sulfur oxides and nitrogen oxides [14]. Unlike pulverized coal combustion fly ash, FBC fly ash exhibits a distinct chemical composition characterized by elevated levels of calcium, sulfur compounds, and unburned carbon [15]. While FBC fly ash is primarily disposed of in landfills [16], there is a growing trend toward incorporating this waste material into polymer composites.

The scientific literature provides various examples of fly ash-reinforced polymer materials, including polyaniline (PANI) [17], geopolymer [18], polyvinyl chloride [19,20], and epoxy resin [21,22], owing to the affordability and widespread availability of fly ash. A PANI/nano-fly ash composite was synthesized by incorporating nano-fly ash (500 nm sample from the Thermal Power Station of Raichur) into PANI, with benzoyl peroxide as the oxidant [17]. A study by Van der Merwe et al. [19] characterized the bulk and surface properties of a South African fly ash sample and developed a method to modify its surface using sodium lauryl sulfate. The feasibility of using the modified fly ash as a replacement for the CaCO_3_ filler in PVC was also investigated. The fly ash was obtained from the Ash Resources PTY Ltd. (Gauteng, South Africa) beneficiation site at Eskom’s Lethabo Thermal Power station. Kumar et al. [20] examined the effects of incorporating fly ash and calcium carbonate as fillers in polyvinyl chloride resin, investigating mechanical properties, such as hardness and compression behavior, as well as moisture absorption behavior. Additionally, Pattanaik et al. [22] investigated the effect of curing conditions on the mechanical properties of fly ash-reinforced epoxy composites. The fly ash filler was obtained from the CPP-2 of Rourkela Steel Plant, Odisha, India. However, limited research has been conducted on the application of FBC fly ash in polyurethane foams. For instance, a study by Kuznia et al. [23] involved producing a polyurethane composite using FBC fly ash, which demonstrated improved mechanical and thermal properties of composite material.

Coal combustion for power and heat generation yields various combustion byproducts, with fly ash being a notable example. The characteristics of fly ash, including the quantity and composition of microspheres present, are influenced by factors such as the combustion method and the fuel properties. Pulverized coal combustion typically yields the greatest amounts of microspheres, which exhibit beneficial attributes like low density, hydrophobicity, and thermal and mechanical stability [24,25]. These microspheres have found widespread industrial applications in areas such as architecture, insulation materials, composite materials, and lightweight concrete. The specific chemical and mineral composition of the microspheres is closely tied to the characteristics of the fuel, the combustion process parameters, the furnace type, and the effectiveness of dust removal systems [26,27]. Due to their availability and versatility, these microspheres can serve as effective modifiers for enhancing the thermal properties, flame retardancy, and overall performance of polyurethane materials [28,29]. The incorporation of these coal combustion byproducts, such as fly ash and microspheres, into polyurethane foam has shown the potential to improve the thermal, mechanical, and physical properties of the composite materials [28,30,31], making them more suitable for a variety of applications, including building insulation, packaging, and lightweight construction [32].

The growing accumulation of waste materials, including non-biodegradable plastics, plant waste, and other discarded products, presents significant environmental challenges [33]. This situation has prompted a global imperative to transition towards a circular economy that prioritizes waste reduction, reuse, and recycling [34]. This transformation involves a fundamental shift from linear “take–make–dispose” models to closed-loop systems where waste is minimized and resources are continuously repurposed. In this context, the utilization of waste materials as fillers in polymer composites represents a promising avenue for sustainable waste management and resource conservation. By incorporating waste materials into composite structures, it is possible to reduce the reliance on virgin materials, mitigate environmental impacts, and potentially enhance the performance characteristics of the resulting composites [35]. This method not only addresses the problem of waste disposal but also contributes to the development of environmentally friendly materials [35]. The utilization of waste materials as fillers in polymer composites presents a sustainable solution that aligns with the principles of the circular economy by transforming discarded materials into valuable resources.

This study examined the effects of two distinct waste materials—fly ash and microspheres—on the properties of rigid polyurethane foam. Fly ash, a byproduct of coal combustion, enhances mechanical strength and thermal stability, while microspheres reduce density and improve insulation. Researchers combined these two components to evaluate whether their mixture could lead to a synergistic effect, further improving the composite material’s properties. To the best of the authors’ knowledge, these two fillers have not been previously mixed together in a single complex system. The researchers analyzed the cellular structure, chemical composition, and mechanical characteristics of the composite materials. Additionally, the foam specimens underwent accelerated aging in a laboratory dryer and outdoor exposure for a specific duration to evaluate their long-term performance and durability under various environmental conditions.

## 2. Materials and Methods

### 2.1. Fabrication of the Polyurethane Foam Composites

In this study, we fabricated rigid polyurethane foam (RPUF) composites by employing the two-component EKOPRODUR PM4032 commercial system (PCC Group, Brzeg Dolny, Poland; specified product characteristics are available online [36]) through a mixing and casting approach. In this investigation, we incorporated two types of modifiers into the polyurethane foam: fly ash (labeled FA) and microspheres (labeled M). The fly ash was derived from the circulating fluidized bed combustion of bituminous coal obtained from a Polish power plant, and it was utilized in its original state without any prior processing or sifting. The initial characterization of the fly ash involved using a variety of techniques detailed in previous research [23,25]. Additionally, the microspheres used in our study were obtained from a coal-fired power plant in Kazakhstan and were isolated from fly ash using the flotation method. These microspheres were incorporated into the foam without any prior preparation or sieving, and they were characterized using a range of methods outlined in prior studies [37].

**Figure 1:**
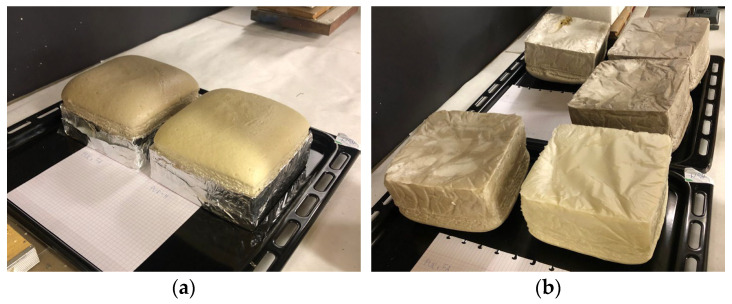
Photographs depicting the polyurethane materials—immediately after the polymer mixtures were poured into the molds (**a**), as well as the final fabricated foam samples upon their removal from the molds (**b**).

To fabricate the composite rigid polyurethane foam samples, we incorporated appropriate amounts of microspheres (5 wt.%) and fly ash (5, 10, and 15 wt.%) into the polyol components. This was achieved by thoroughly stirring the mixture using a laboratory stirrer at a high speed of 4500 rpm for one minute. The polyol and isocyanate components (weight ratio of 100:110) were then combined using the same stirring method for an additional minute before being poured into rectangular molds measuring 20 × 20 × 5 cm (Figure 1a). The filled molds were subsequently placed under a fume hood to allow the foams to set and cure. After a 48 h period, the foams were demolded and then rested under the fume hood for five more days to ensure the complete removal of any unreacted isocyanate (Figure 1b). A total of four different material types were produced. The resulting composite foams were designated as 5M5FA, 5M10FA, and 5M15FA, while the unmodified rigid polyurethane foam was labeled as PUR.

### 2.2. Evaluation and Analysis of the Foam Samples

To examine the cellular structure of the stabilized rigid polyurethane foam, we selected and analyzed samples. Using a Keyence VHX-900F microscope (Keyence, Osaka, Japan), we captured optical microphotographs of the samples. Prior to imaging, the foam samples were cross-sectioned with a sharp blade, yielding 5 mm thick slices. For each material type, 3 slices were used for picture acquisition. From each slice, one microstructure image was taken from a different part. From each image, 10 measurements were made, resulting in 30 individual observations per sample. The analysis of these microphotographs employed ImageJ software (version 1.48v) to calculate the average values and standard deviations for various structural parameters, including strut thickness, horizontal Feret diameter, and vertical Feret diameter.

The microstructure of the fabricated foams was analyzed using scanning electron microscopy (Nova NanoSEM 200; FEI Company, Hillsboro, OR, USA). Specimens were prepared for imaging by coating them with a thin layer of gold to enhance image quality. The samples were then imaged in the secondary electron mode with an accelerating voltage of 2–5 kV and a spot size of 3.0.

Fourier-transform infrared spectroscopy was employed using a Tensor 27 spectrometer and OPUS 7.2 software (Bruker Optics, Billerica, MA, USA). The absorbance mode was utilized to acquire spectra in the range of 4000–400 cm^−1^, with 64 scans averaged at a resolution of 4 cm^−1^. Sample preparation involved the use of the KBr pellet method. The materials examined included unmodified RPUF, fly ash, microspheres, and 5M15FA composite foam. Fly ash and microspheres were used as obtained. The foamed samples were ground, and the fine powder was mixed with KBr. Subsequently, the mixture was pressed into a pellet for analysis. These FTIR spectra were subsequently analyzed to determine the chemical structure and composition of the RPUF composite material.

The apparent densities of the composite polyurethane foam samples were determined in accordance with the ASTM D1622-03 standard [38]. To measure the densities, the polymeric foam samples were weighed, and their dimensions were carefully recorded. The reported results represent the average of 5 individual density measurements for each sample, accompanied by the corresponding standard deviation to provide a measure of the variability in the data.

The mechanical properties of the RPUF composite were evaluated using a Zwick 1435 universal testing machine (Zwick Roell, Ulm, Germany) equipped with a 5 kN load cell. Cylindrical foam samples with 30 mm diameters and 12 mm heights were subjected to compression testing at a rate of 2 mm min^−1^ until they reached a 75% deformation level. The compressive strength (*R_m_*) and Young’s modulus (*E*) were then calculated from the resulting stress–strain curves. The reported values represent the average of five individual tests along with the corresponding standard deviations to provide a measure of the variability in the data. This comprehensive evaluation allowed for a thorough characterization of the mechanical properties of the RPUF composite material.

### 2.3. Evaluation of Aging Behavior

To assess the thermal resilience and stability of the composite rigid polyurethane foam under normal environmental conditions, the researchers cut the foam material into cubic blocks measuring 4 × 4 × 4 cm. The groups of foam underwent accelerated aging by being placed in a dryer with forced air circulation at a temperature of 120 °C for 48 h. Additionally, the RPUF composite samples were exposed to natural ambient conditions by being kept outdoors for 21 days without direct sunlight exposure. During this outdoor exposure, the temperature fluctuated between 1 and 8 °C, the atmospheric pressure ranged from 976 to 1040 hPa, and the humidity varied between 70 and 86%. After these aging treatments, an analysis was performed to comprehensively evaluate the cellular structure and mechanical performance of the samples. The experimental parameters were chosen based on existing studies in the scientific literature that have investigated the aging behavior of similar polymer composite materials [39,40,41].

## 3. Results

### 3.1. Cellular Structure and Microstructure

**Figure 2:**
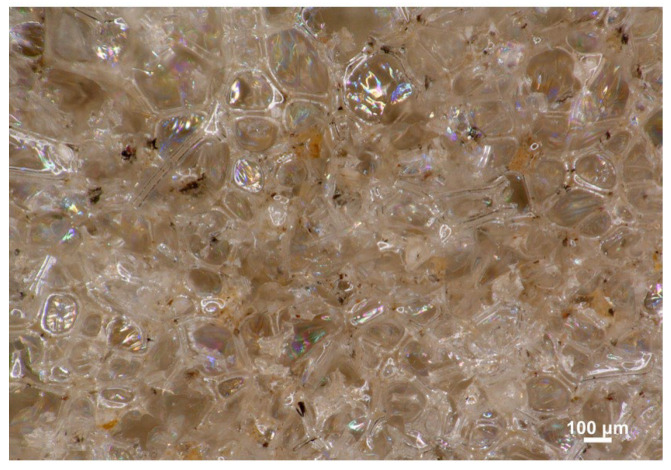
Optical micrograph of the 5M15FA composite foam. The magnification used was 150×.

**Figure 3:**
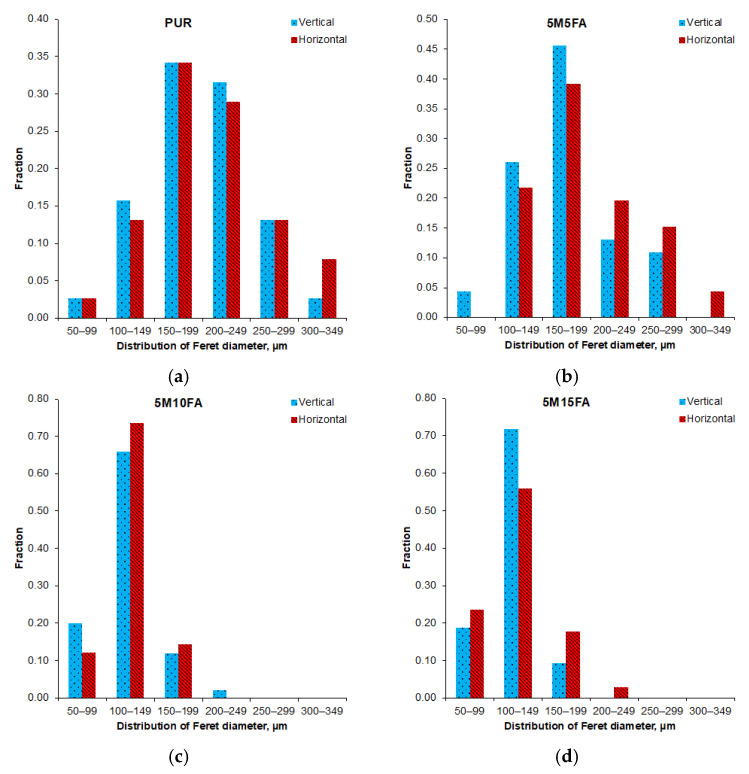
Histograms depicting the Feret diameters for the pristine polyurethane foam (**a**) and the composite foam groups with varying filler contents (5M5FA (**b**), 5M10FA (**c**), and 5M15FA (**d**)).

Optical microscopic analysis of the 5M15FA composite foam sample, as shown in Figure 2, reveals the typical cellular structure characteristic of polyurethane foams. The micrographs of all the composite RPUF samples demonstrate a predominance of pentagonal cells with regular, well-defined shapes. The cellular morphology parameters are further detailed in Table 1. The vertical and horizontal Feret diameters exhibited similar measurements, indicating the absence of a dominant growth direction. As the modifier content increased from 5 to 15%, the vertical Feret diameters decreased, ranging from 120 to 175 μm, while the horizontal Feret diameters ranged from 130 to 190 μm. The cell size remained consistent across varying filler concentrations, mostly falling within the approximate range of 100 to 200 μm, as depicted in Figure 3. The thickness of the cell struts showed no significant variation due to the filler content, maintaining stability at around 18 μm. Moreover, the fillers demonstrated an even dispersion pattern throughout the polymer matrix.

**Figure 4:**
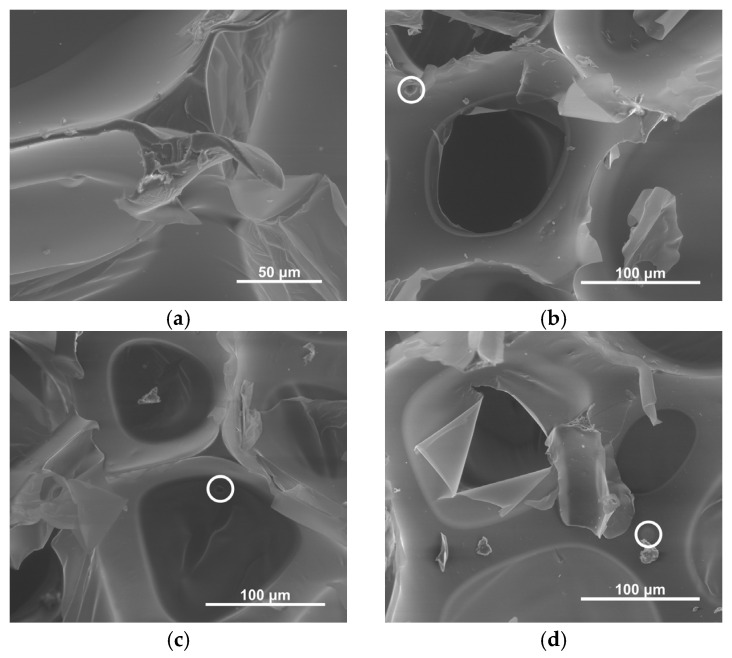
Scanning electron micrographs of PUR (**a**), 5M5FA (**b**), 5M10FA (**c**), 5M15FA, and (**d**) composite foams. Filler particles were denoted with encircled regions. The magnification used was 1000×.

The scanning electron micrographs revealed that the cellular structure of the composite RPUF was well developed (Figure 4). The polymer matrices exhibited uniformity, with the filler particles (microspheres and fly ash) evenly dispersed throughout and no accumulations detected. Since the SEM samples were coated with gold to enhance the image quality, the test results could not be used to quantify the cellular parameters. Instead, these important microstructural characteristics were determined through detailed optical microscopy observations. The inclusion of both microspheres and fly ash in the composite RPUF materials did not significantly impact the overall cellular structure or morphology of the polyurethane foam.

### 3.2. Characterization of Chemical Composition and Mechanical Behavior

**Figure 5:**
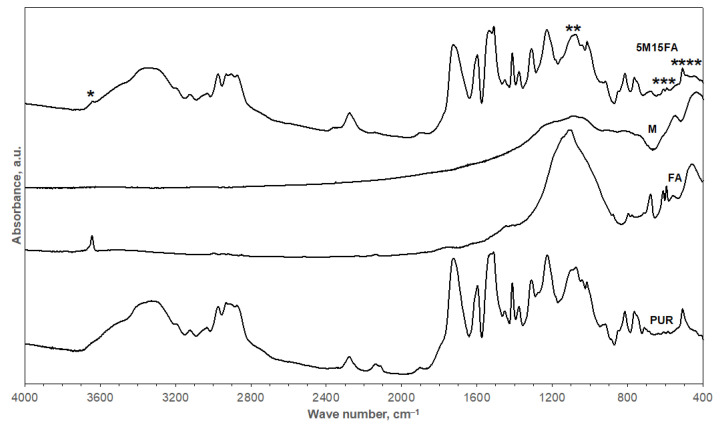
The FTIR spectra of the pristine polyurethane foam (PUR), microspheres (M), fly ash (FA), and polyurethane foam composite containing 5 wt.% M and 15 wt.% FA (5M15FA). Bands indicating the presence of fillers within the polymer matrix in composite 5M15FA were marked with ‘*’, ‘**’, ‘***’, and ‘****’.

The FTIR spectrum of the polyurethane foam (Figure 5; bottom curve) revealed absorption bands corresponding to various components of the polyurethane matrix. Stretching vibrations of N–H groups were observed at around 3350 cm^−1^. Symmetric and asymmetric stretching movements of the C–H bonds within aliphatic chains appeared in the range of 3000–2800 cm^−1^. Vibrations associated with the phenyl ring in the isocyanate structure (also unreacted) occurred at 2300, 2150, and 1600 cm^−1^. C=O bonds exhibited stretching vibrations at 1700 cm^−1^. The bending vibrations of N–H groups were located at a wavenumber of 1500 cm^−1^. Vibrations linked to the CH_2_ and CH_3_ groups occurred at a wavenumber of 1400 cm^−1^. The stretching vibration of C–N bonds was present at 1300 cm^−1^. The stretching vibration of C–O bonds appeared at around 1200 cm^−1^. Vibrations associated with ether linkages within the polyol structure were in the range of 1100–1000 cm^−1^. The vibrations related to C–C bonds and aromatic rings within the isocyanate structure occurred in the range of 900–700 cm^−1^ [28,42,43].

Two central curves in Figure 5 illustrate the FTIR spectra of the fly ash (FA) and microspheres (M). Based on the X-ray diffraction evaluation, the FA sample contained crystalline mullite and quartz phases [25]. Meanwhile, M consisted primarily of an amorphous phase. The identified mineral phases included mullite, corundum, and quartz [37]. The close resemblance in their crystalline composition led to similar FTIR spectra being recorded for both fillers. Bands linked to the silica (quartz) displayed clear characteristics in both FA and M spectra. The band at approximately 1100 cm^−1^ was assigned to the asymmetric stretching vibrations of the Si–O group, while the band at 460 cm^−1^ was associated with the vibration of O–Si–O in silicate tetrahedra. Additionally, the band appearing at around 800–700 cm^−1^ indicated the stretching vibration of Si–O–Si bridges, and a band located at about 550 cm^−1^ was linked to the presence of Al in tetrahedral positions. A weak band at 3650 cm^−1^ corresponded to the presence of hydroxyl groups within the ash structure [44,45].

The FTIR spectrum of the 5M15FA composite (Figure 5; upper curve) was highly similar to that of the unmodified polyurethane foam. The characteristic absorption bands associated with both the polyurethane and the fly ash/microsphere fillers were observed in comparable wavenumber regions, with only minor variations in the 5M15FA spectrum. These subtle differences suggest the presence of fillers within the polymer matrix. Specific bands were observed at (*) 3650 cm^−1^, corresponding to hydroxyl groups within the ash structure; (**) 1100 cm^−1^, which was assigned to the asymmetric stretching vibrations of the Si–O group; (***) 550 cm^−1^, which was linked to aluminum in tetrahedral positions; and (****) 460 cm^−1^, which was associated with the vibration of O–Si–O in silicate tetrahedra. No additional bands corresponding to other chemical compounds were detected.

**Figure 6:**
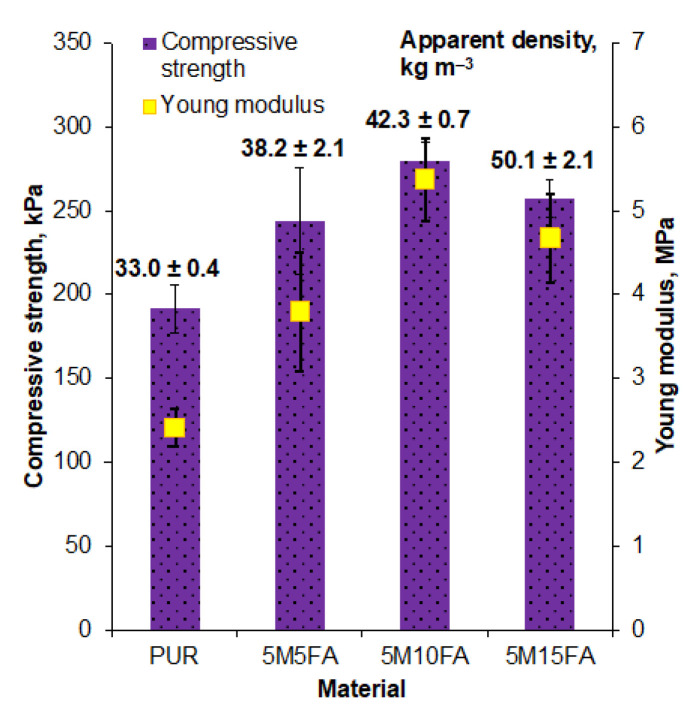
Foam density and mechanical property data.

The addition of fillers, such as microspheres and fly ash, consistently increased the apparent density of rigid polyurethane foam (Figure 6), as documented in previous studies. The 5M15FA composition, containing 5% microspheres and 15% fly ash by weight, ultimately reached an apparent density of approximately 50 kg m^−3^.

The compressive strength and Young’s modulus values obtained from the mechanical testing are presented in Figure 6. The inclusion of both fillers, up to 15% by weight, resulted in an increase in both the compressive strength and Young’s modulus compared to the unmodified sample. However, the mechanical properties calculated for the 5M15FA composition were slightly lower compared to the other composites, suggesting the potential overloading of the polymer matrix with filler particles.

### 3.3. Durability Assessments

**Figure 7:**
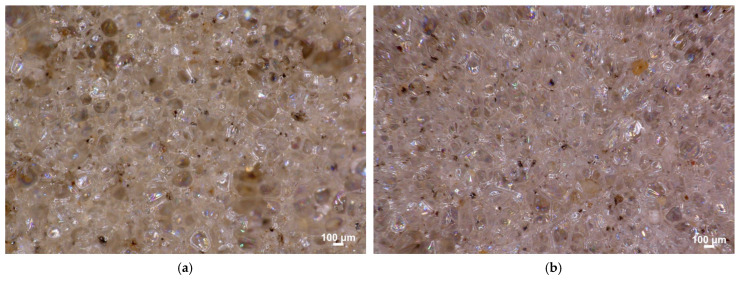
Optical micrographs of the 5M15FA polyurethane foam composite following accelerated aging in a laboratory dryer (**a**) and exposure to ambient conditions (**b**). The samples were examined at a magnification of 100×.

Optical micrographs of the 5M15FA polyurethane foam composite were analyzed after subjecting the material to accelerated aging in a laboratory dryer and exposure to ambient conditions, as depicted in Figure 7 and the corresponding figures, respectively. The examined samples exhibited a characteristic cellular structure commonly observed in polyurethane foam, with predominantly pentagonal cells displaying a uniform morphology.

**Figure 8:**
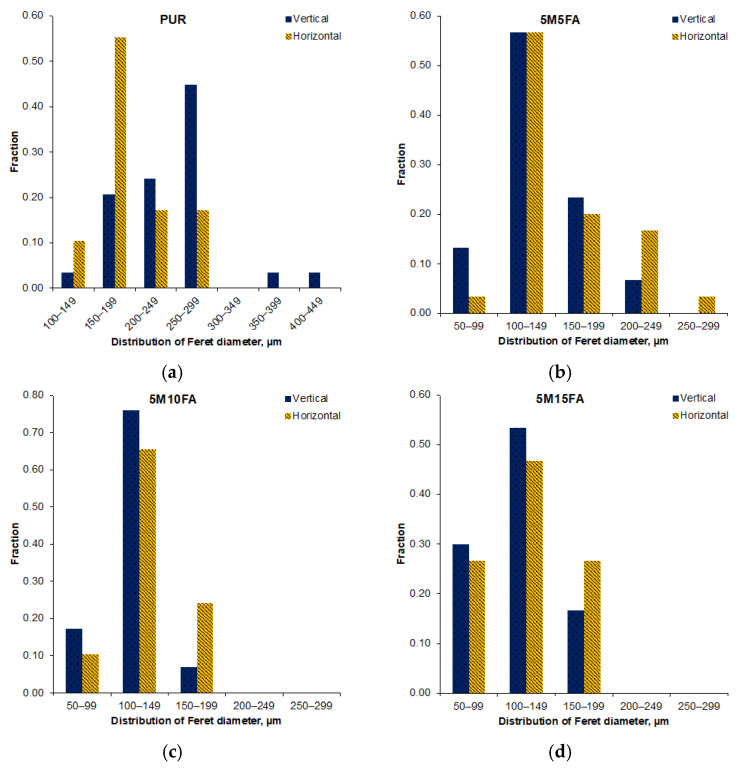
The Feret diameter distributions of the pristine polyurethane foam (PUR) (**a**) and the 5M5FA (**b**), 5M10FA (**c**), and 5M15FA (**d**) composite foams after accelerated aging in a laboratory dryer.

**Figure 9:**
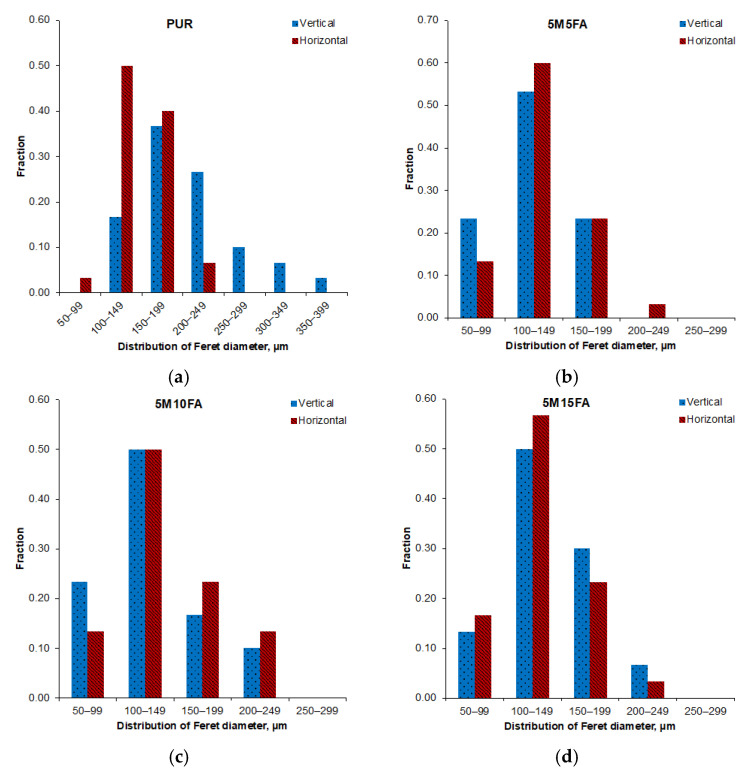
The histograms depicting the Feret diameters for the pristine polyurethane foam (**a**) and the composite foams with varying filler contents (5M5FA (**b**), 5M10FA (**c**), and 5M15FA (**d**)) after exposure in ambient conditions.

Table 2 and Table 3 present the cell measurements obtained after subjecting the materials to accelerated aging in a controlled environment and natural ambient conditions, respectively. The strut thickness remained consistent across both scenarios, as did the diameter distribution (Figure 8 and Figure 9). Compared to their initial state, the Feret diameters of the pristine PUR foam increased following simulated aging in the controlled environment. In contrast, the composite samples exhibited comparable dimensions after undergoing accelerated aging in a laboratory setting. However, subsequent exposure to natural ambient conditions led to an approximately 10% increase in both diameters for the composite material samples.

**Figure 10:**
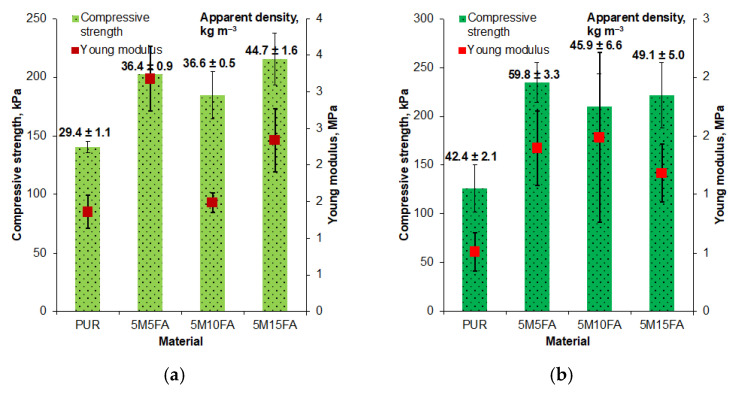
Density of different types of foam and mechanical performance parameters after accelerated aging in laboratory dryer (**a**) and after exposure to ambient conditions (**b**).

Accelerated aging in a controlled laboratory environment had little impact on the apparent densities of the materials (Figure 10). However, a slight decrease was observed for the 5M10FA and 5M15FA composite samples. In contrast, exposure to natural ambient conditions led to an increase in the densities of all the specimens, with the 5M15FA composite displaying the smallest change (Figure 10).

All the examined materials exhibited a decline in mechanical performance following the aging experiments. Pristine polyurethane foam experienced the most significant decreases in compressive strength and Young’s modulus. In contrast, the composite materials demonstrated a less pronounced reduction in these mechanical properties.

## 4. Discussion

The optical micrographs of the composite RPUF displayed a typical cellular structure for polyurethane foam, characterized by predominantly pentagonal cells with uniform shapes. The vertical and horizontal Feret diameters exhibited comparable measurements, indicating the absence of a dominant growth direction. The literature extensively discusses how filler particles act as nucleation sites during the formation of polyurethane cellular structures, leading to a reduction in cell diameters [46,47,48,49]. The cell size remained consistent across varying filler concentrations. Additionally, the thickness of the cell struts showed no significant variation due to the filler content. The fillers were observed to be evenly dispersed throughout the polymer matrix. The consistent cell size and strut thickness across different filler concentrations suggest that the addition of fillers did not significantly alter the overall cellular morphology of the polyurethane foam but rather influenced the specific dimensions of the Feret diameters.

The FTIR analysis of the polyurethane foam composite revealed absorption bands corresponding to the various components of the polyurethane matrix. The similar crystalline composition of the fly ash and microsphere fillers resulted in comparable FTIR spectra being recorded for both. The FTIR spectra of the fly ash and microsphere fillers displayed clear characteristics associated with silica. The resemblance in their crystalline structure led to these similar FTIR profiles. Furthermore, the FTIR spectra provided additional insights into the chemical composition and structure of the fly ash and microsphere fillers, which can be valuable to understanding their interactions and effects within the polyurethane foam composite. The FTIR spectrum of the 5M15FA composite exhibited significant overlap with the unmodified polyurethane foam spectrum, likely due to the predominance of the polymer matrix in the composite material. The characteristic absorption bands associated with both the polyurethane and the fly ash/microsphere fillers were observed in similar wavenumber regions, with only minor variations in the 5M15FA spectrum. These minor differences suggest the presence of the fillers within the polymer matrix. Importantly, no additional bands corresponding to other chemical compounds were detected, indicating the absence of any chemical bonding between the polymer and the fillers, which suggests that the incorporation of the fillers did not significantly alter the chemical composition of the polyurethane foam.

Numerous prior studies have consistently reported an increase in the apparent density of rigid polyurethane foam upon the incorporation of fillers [50,51,52]. This trend has been widely documented across various research publications.

The incorporation of both fillers, up to 15% by weight, led to enhanced compressive strength and Young’s modulus compared to the unmodified polyurethane foam. This suggests potential beneficial interfacial interactions between the polymer matrix and fillers, as well as a uniform distribution of the fillers within the polymer matrix. However, the mechanical properties calculated for the 5M15FA composition were slightly lower than the other composites, indicating the potential overloading of the polymer matrix with filler particles. These findings align with the conclusions drawn in previous related studies [28,43,53].

The examined samples displayed a characteristic cellular morphology commonly observed in polyurethane foam, featuring predominantly pentagonal cells with a uniform structure. Notably, the simulated aging treatments seemed to have minimal impact on the cellular architecture of the materials at this stage of the investigation. The findings suggest that the polyurethane foam composite maintained its structural integrity and stability even after its exposure to accelerated aging conditions, which is a positive indication of the material’s durability and potential for long-term performance.

The strut thickness exhibited consistent measurements across both aging regimes, and the diameter distribution was likewise maintained. Relative to their initial state, the Feret diameters of the unmodified polyurethane foam increased after simulated aging in the controlled environment. Conversely, the composite samples displayed comparable dimensions following accelerated aging in a laboratory setting. However, subsequent exposure to natural ambient conditions led to an increase in both diameters for the composite material samples.

The accelerated aging in a controlled laboratory environment did not significantly impact the apparent densities of the materials. However, a subtle decrease was observed for the composites containing 10% and 15% filler by weight (5M10FA and 5M15FA, respectively). This suggests that the filler content may have contributed to a slight reduction in apparent density under accelerated aging conditions. Conversely, when exposed to natural ambient conditions, the densities of all the specimens increased, with the 5M15FA composite exhibiting the smallest change. This indicates that the environmental factors encountered during natural aging had a more pronounced effect on the apparent density, potentially due to interactions between the polymer matrix, fillers, and the surrounding environment.

The materials examined showed a decline in mechanical performance after aging. The unmodified polyurethane foam had the greatest decrease in compressive strength and Young’s modulus. In contrast, the composite materials experienced a less significant reduction in these mechanical properties. This indicates that adding microspheres and fly ash fillers improved the materials’ durability and resistance to aging effects.

Overall, exposure to ambient conditions had a more noticeable effect on the mechanical parameters compared to accelerated aging in the controlled laboratory environment. The findings of the aging evaluations align with the conclusions drawn in previous related studies [39,40,41], which have also reported on the impact of aging on the properties of polyurethane-based composite materials.

## 5. Conclusions

This study aimed to investigate the influence of fillers, including fly ash and microspheres, on the chemical structure, mechanical characteristics, and aging behavior of composite rigid polyurethane foam materials. FTIR analysis uncovered distinctive absorption bands corresponding to various components of the polyurethane matrix as well as the incorporated fillers. The presence of fillers in the RPUF composite led to observable changes in specific absorption bands, indicating the incorporation of fillers within the polymer matrix without significant chemical bonding. Furthermore, the mechanical testing demonstrated that the inclusion of fillers resulted in improved compressive strength and Young’s modulus compared to the unmodified RPUF sample. However, excessive filler content, as observed in the 5M15FA composite, led to a slight decline in mechanical properties, suggesting the potential overloading of the polymer matrix with filler particles.

Additionally, aging experiments revealed that simulated accelerated aging and exposure to natural ambient conditions had minimal impact on the cellular structure of the RPUF materials. Nevertheless, the aging experiments did result in the deterioration of mechanical performance, with exposure to ambient conditions exhibiting a more pronounced effect on the mechanical parameters compared to accelerated aging in a laboratory setting.

In conclusion, the research findings suggest that the addition of fillers, up to a certain threshold, can enhance the mechanical properties of RPUF materials without significantly affecting their cellular structure. However, further optimization of the filler content is necessary to maintain the mechanical integrity of the composite materials during aging. These results contribute to an understanding of the potential applications of RPUF composites in various engineering and construction fields, where their improved mechanical properties and durability can be leveraged.

## Figures and Tables

**Table 1:** Key cellular morphology parameters of the foam samples.

Sample Name	Vertical Feret Diameter, μm	Horizontal Feret Diameter, μm	Strut Thickness, μm
PUR	193.4 ± 15.3	207.4 ± 15.7	19.8 ± 1.2
5M5FA	175.2 ± 12.7	192.5 ± 13.1	18.7 ± 0.6
5M10FA	124.5 ± 6.7	125.5 ± 6.0	17.0 ± 0.5
5M15FA	120.6 ± 7.4	129.9 ± 10.5	18.0 ± 0.5

**Table 2:** The cellular morphology parameters of the foam groups after accelerated aging in a laboratory dryer.

Sample Name	Vertical Feret Diameter, μm	Horizontal Feret Diameter, μm	Strut Thickness, μm
PUR	248.7 ± 0.9	198.0 ± 0.8	18.4 ± 0.7
5M5FA	134.6 ± 0.7	146.1 ± 0.7	20.0 ± 0.7
5M10FA	118.7 ± 0.6	130.0 ± 0.7	17.7 ± 0.6
5M15FA	119.8 ± 0.6	128.3 ± 0.7	17.6 ± 0.4

**Table 3:** The cellular characteristics of the foam groups following exposure to natural ambient conditions.

Sample Name	Vertical Feret Diameter, μm	Horizontal Feret Diameter, μm	Strut Thickness, μm
PUR	208.1 ± 0.8	150.7 ± 0.7	21.0 ± 1.0
5M5FA	129.0 ± 0.7	132.8 ± 0.7	20.3 ± 1.0
5M10FA	134.4 ± 0.7	142.4 ± 0.7	19.2 ± 1.1
5M15FA	141.1 ± 0.7	132.8 ± 0.7	21.2 ± 1.1

## Data Availability

The original contributions presented in this study are included in the article. Further inquiries can be directed to the corresponding author.

## Abbreviations

The following abbreviations are used in this manuscript:

RPUF	Rigid polyurethane foam
PCC	Pulverized coal combustion
FBC	Fluidized bed combustion
SEM	Scanning electron microscopy
FTIR	Fourier-transform infrared spectroscopy
